# Low-Grade Malignant Peripheral Nerve Sheath Tumor: A Case Report of Exceptional Parapatellar Location in the Knee

**DOI:** 10.7759/cureus.52739

**Published:** 2024-01-22

**Authors:** Mohammed Barrached, Achraf Tebbaa El Hassali

**Affiliations:** 1 Department of Traumatology and Orthopedic, Mohamed First University, Mohammed VI University Hospital, Oujda, MAR

**Keywords:** malignant tumor of the peripheral nerve sheaths, nerve tumors, knee, exceptional location, diagnosis

## Abstract

Malignant tumors of the peripheral nerve sheaths are uncommon, constituting a small percentage, typically ranging from 2% to 5% of soft tissue sarcomas. Etiological diagnosis is often difficult but is guided by imaging and confirmed by histopathological and immunohistochemical examination. We report a case of a 46-year-old woman admitted for management of a mass in the medial parapatellar region of the right knee. Her medical history included a burn to the right leg five years ago and a previously undocumented resection of a medial parapatellar tissue mass in the right knee. Radiological examination showed a deep and superficial soft tissue mass in the medial soft tissue of the right knee opposite the patella, with no hemorrhagic components and heterogeneous enhancement after injection of gadolinium. Histopathology confirmed the diagnosis of a low-grade peripheral nerve sheath tumor, and a thoracic-abdominal-pelvic CT scan was performed, which was normal. Treatment consisted of a simple carcinological resection and local radiotherapy. No local recurrence was noted after one year of follow-up.

## Introduction

The peripheral nervous system is defined as all nerve tissue outside the brain and spinal cord. Malignant peripheral nerve sheath tumors (MPNST) are spindle-shaped sarcomas with nerve differentiation and account for 2% to 5% of soft tissue sarcomas [1,2]. MPNSTs arise from the nerve sheaths of myelinated nerves, usually the spinal and intercostal nerves. We report a case of an exceptional parapatellar localization of a malignant tumor of the peripheral nerve sheaths.

## Case presentation

A 46-year-old female patient with a history of burns to the right leg five years ago, a history of resection of a medial parapatellar tissue mass in the right knee that had been evolving for four years, not sent for anatomopathological examination, referred to our department for management of a recurrence of the mass after eight months.

**Figure 1:**
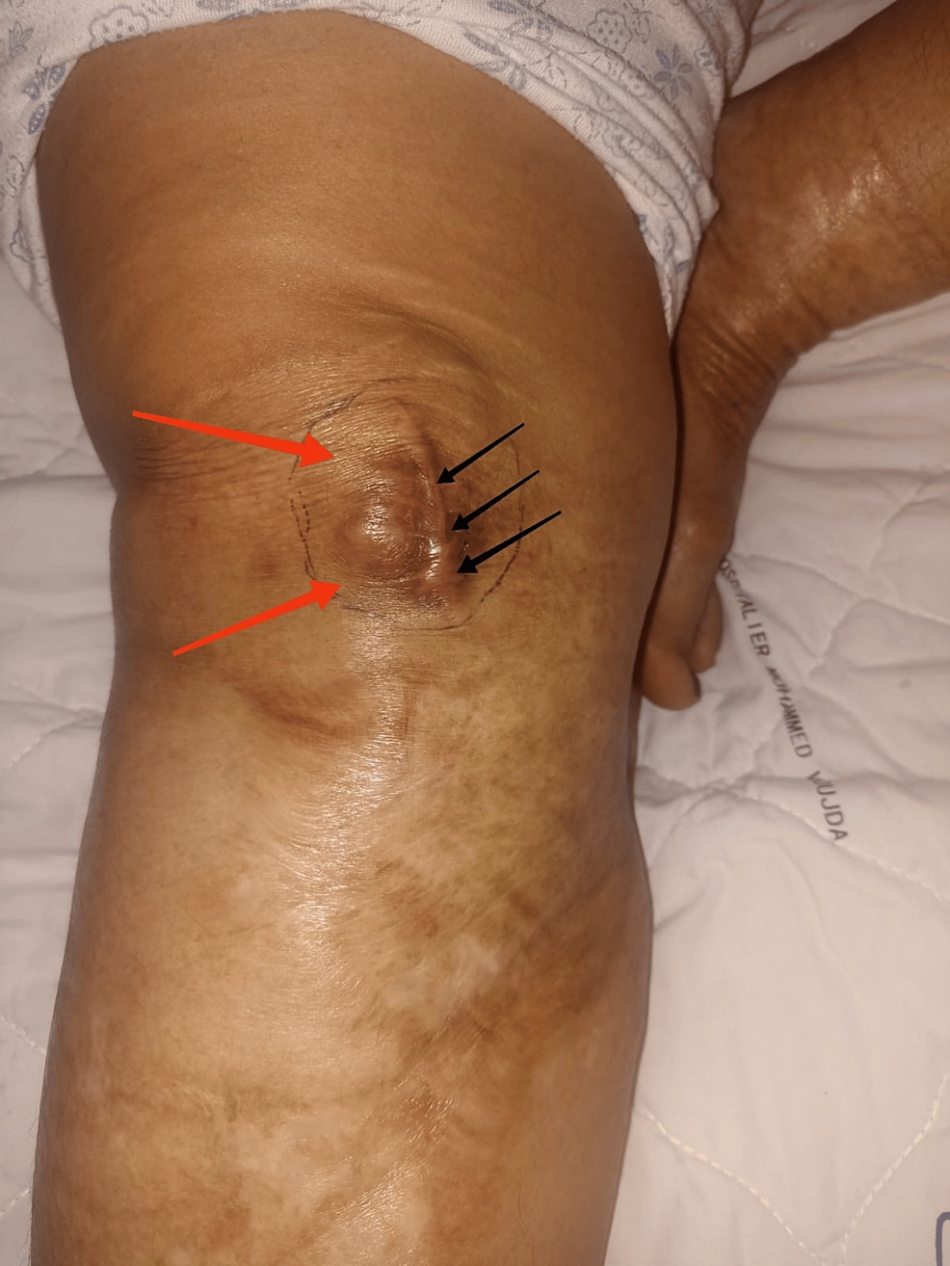
Clinical image shows a medial parapatellar mass in the right knee (red arrows), keloid scarring from the previous approach (black arrows), with no inflammatory signs adjacent to the mass.

Clinical examination revealed a keloid scar from the previous approach, with no inflammatory signs adjacent to it, and a medial parapatellar mass on the right knee that was tender to palpation, ovoid, hard, and fixed in relation to the deep and superficial planes. The mass was 4 cm long, and the patient reported a notion of curvature on the medial aspect of the right knee (Figure 1). There was no inguinal adenopathy, and the patient was apyretic and in good general health.

**Figure 2:**
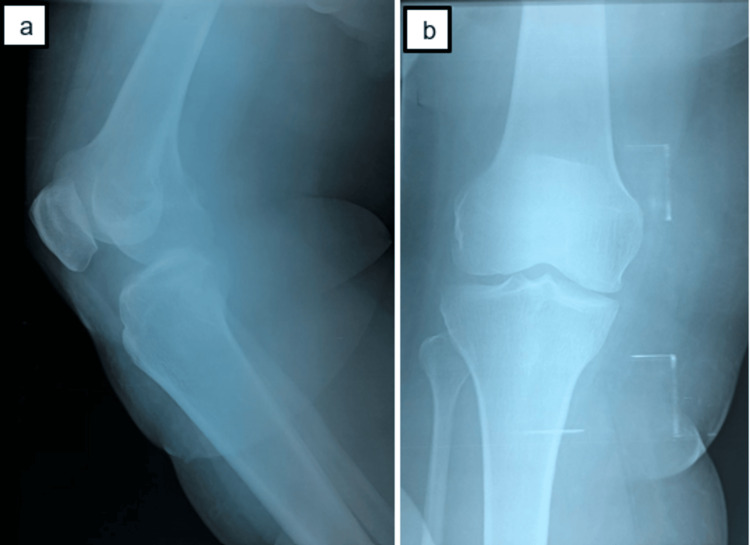
X-ray of the right knee (side view (a) and front view (b)) shows no bone damage.

**Figure 3:**
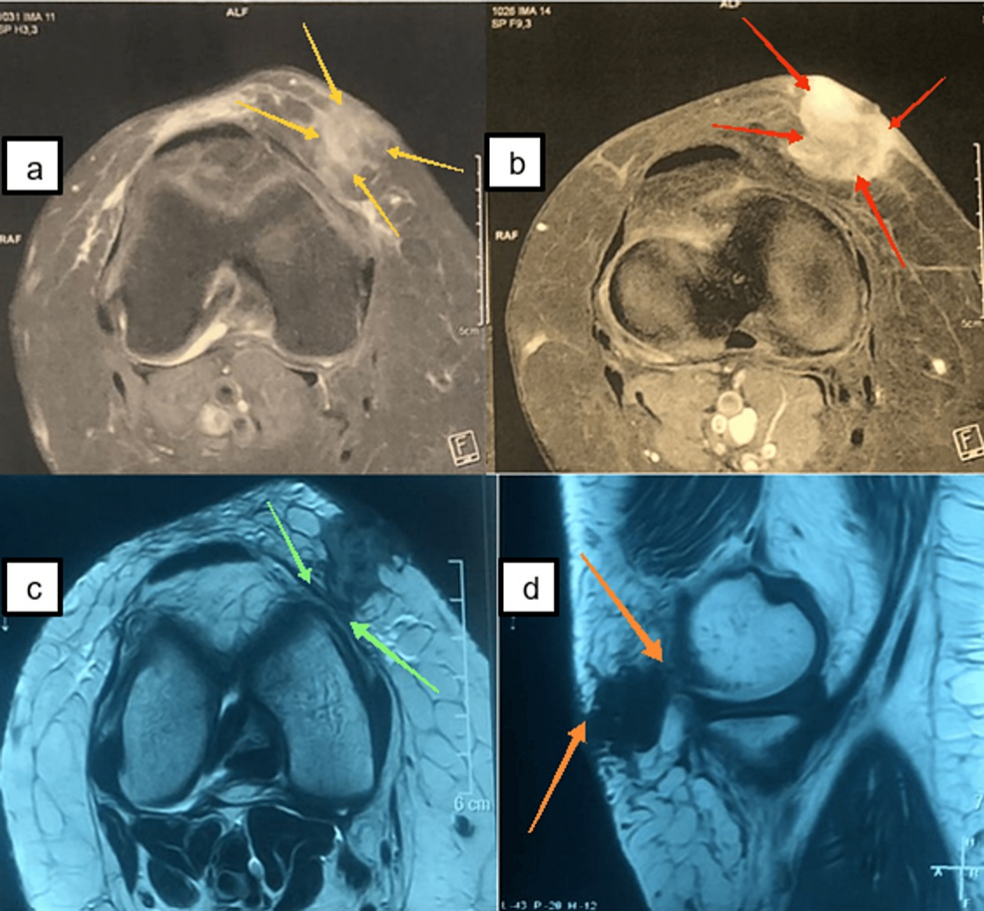
MRI of the right knee shows an oval tissue mass in the medial soft tissues of the right knee opposite the patella (yellow arrows) with deep and superficial development (orange arrows), containing no hemorrhagic components with heterogeneous enhancement after injection of gadolinium (red arrows) and no osteoligamentous, meniscal, or tendon abnormalities in the knee (green arrows). MRI, magnetic resonance imaging

X-ray of the knee was normal (Figure 2). Magnetic resonance imaging (MRI) of the knee showed a tissue formation in the medial soft tissues of the right knee opposite the patella with deep and superficial development, roughly oval-shaped, intermediate hyposignal in T1, intermediate signal in T2, and discrete diffusion hypersignal, heterogeneously enhancing after injection of Gadolium, not fading on the STIR sequence, no hemorrhagic component, in contact with the medial collateral ligaments, no joint effusion, and no osteoligament, meniscus, or tendon abnormalities in the knee (Figure 3).

The biological examination was unremarkable. The patient presented with an unexplained mass around the knee, the diagnosis of which is sometimes difficult. We therefore performed a biopsy of the mass to analyze its histological type in order to rule out a tumor pathology.

**Figure 4:**
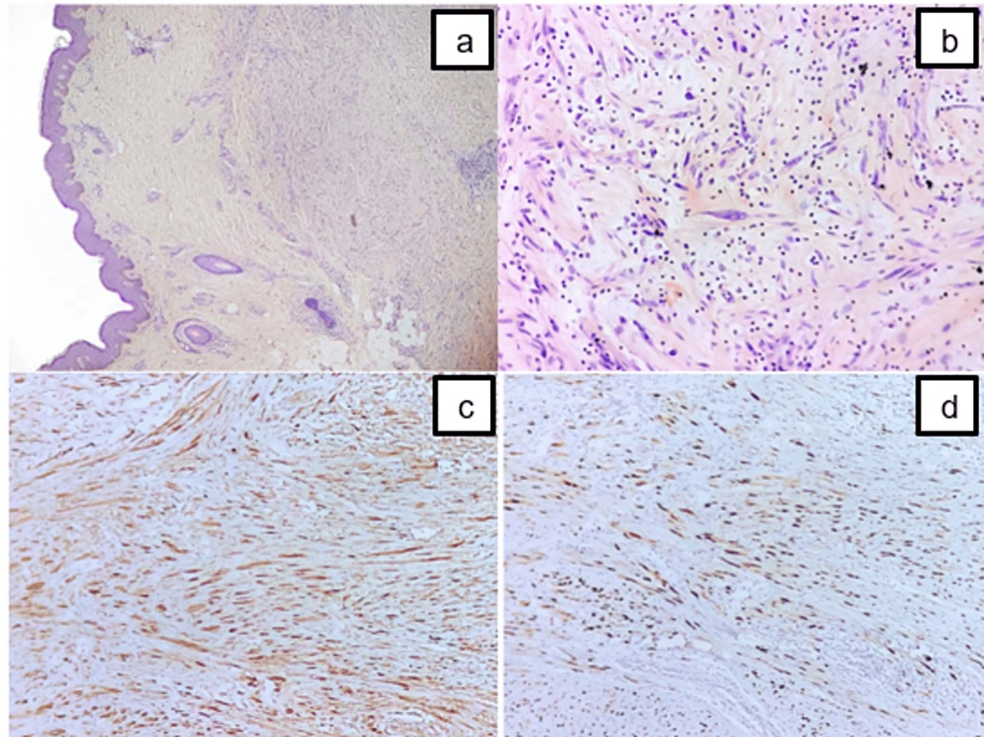
Microscopic examination of the lesion shows diffuse infiltration of the deep dermis by a spindle cell tumor ((a) H&E x40). Tumor cells are spindle-shaped, exhibiting mild to moderate atypia, and surrounded by a fibrous stroma ((b) H&E x400). Immunohistochemical staining demonstrates a strong but limited positivity of tumor cells for both S100 (c) and SOX10 (d). H&E, hematoxylin and eosin

The anatomopathological study revealed a histological and immunohistochemical appearance in favor of a low-grade peripheral nerve sheath tumor with a proliferative index assessed by ki67 of 20% (Figure 4). In search of a secondary location, a thoraco-abdomino-pelvic CT scan was performed, which was normal.

**Figure 5:**
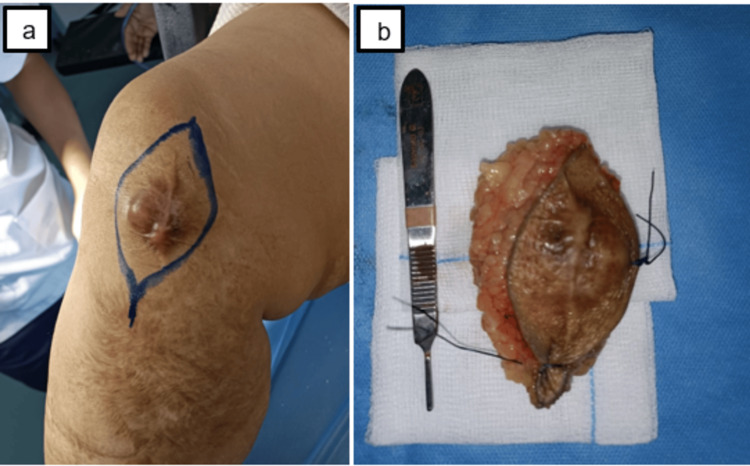
Intraoperative image shows the limits of the skin incision (a) and the mass after resection (b).

Following the multidisciplinary consultation meeting (RCP), the therapeutic decision was a simple carcinological resection of the mass. The patient underwent surgical resection of the mass; under spinal anesthesia and pneumatic tourniquet, a new approach was used, taking the old surgical scar. Cutaneous and subcutaneous incisions were made, and we found a hard, brownish mass. The mass was resected in its entirety while respecting the tendon structures of the knee (Figure 5). Then plane-by-plane closure of the incision without recourse to the skin augmentation procedure.

Given the strong suspicion of a local recurrence after the first resection of the mass (undocumented), the patient underwent five sessions of local radiotherapy. No local recurrence was observed after one year of follow-up.

## Discussion

Peripheral nerve sheath tumors account for between 2% and 5% of soft tissue sarcomas, although they have specific relative frequencies and certain characteristics related to their location [1-3]. Nerve sheath tumors are tumors of adults and nerve cell tumors are tumors of children [3,4]. Nerve sheath tumors are formed by the proliferation of nervous elements in the sheaths of peripheral nerves: Schwann cells, perineural cells, and fibroblasts. They develop at the expense of spinal nerves in 90% of cases, and secondarily at the expense of spinal roots, communicating branches, or intercostal nerves, and are most often located in the posterior paravertebral mediastinum close to the neuraxis [3-5]. The location of the tumor varies according to its position on the nerve pathway. The originality of our case lies in the location of the nerve tumor in the parapatellar region of the knee. However, the nerve of origin could not be determined intraoperatively. The second particularity of our case is the malignancy of the nerve tumor.

In general, what brings the patient to the clinic is the discovery of an ovoid swelling, the contact of which often provokes dysesthesia in the form of an electric discharge in a nerve trunk. More rarely, they consult us for paresthesia or numbness in the distal territory of a nerve trunk, and exceptionally for motor discomfort. MRI, which is more informative, is the examination of choice for diagnosing peripheral nerve tumors (PNT). It is very easy to diagnose PNT on T1, T1 + gadolinium, and T2 sequences, and above all because of its multi-planar capabilities. Some teams use a special "neurography" sequence. These rapid FSE T2 acquisition sequences in very thin sections, combined with a Fat-Sat type fat saturation technique, provide images of the nerve trunk and the tumor [6].

The surgical procedure usually begins with a biopsy combined with an extemporaneous examination to confirm the suspected malignant diagnosis. Open surgery is still indicated for malignant tumors requiring extended resection in one or two stages [3,4,7]. In our case, extended resection was carried out to limits considered anatomopathologically to be free of tumor infiltration. MPNST are very aggressive, with a high rate of recurrence after excision and of distant metastases. Their prognosis is poor, with a five-year survival rate of 50% [3,8,9]. The poor prognostic factors described in the literature are a tumor larger than 5 cm, incomplete resection, and underlying neurofibromatosis [3,10].

Treatment with radiotherapy and/or chemotherapy is of limited value, and radiotherapy is often recommended for high-grade lesions or tumors larger than 5 cm [11]. The long-term result of radiotherapy is excellent local control [12,13]. However, adjuvant radiotherapy is not beneficial for the survival of MPNST, although in some studies adjuvant radiotherapy has been used to reduce tumor size to make surgery possible [10]. Unlike surgical resection and radiotherapy, chemotherapy is generally limited to the treatment of metastatic MPNST or to patients whose tumors are not resected [14].

## Conclusions

Peripheral nerve sheath tumors are characterized by significant morphological and phenotypic variability. This variability is a source of problems and difficulties in terms of diagnosis hence the importance of having a precise diagnosis guided by a clinical and radiological examination and confirmed by histological evidence before beginning definitive treatment. The particularity of our observation is the exceptional location parapatellar of the knee.

## References

[1] Lewis JJ, Brennan MF (1996). Soft tissue sarcomas. Curr Probl Surg.

[2] Ng VY, Scharschmidt TJ, Mayerson JL, Fisher JL (2013). Incidence and survival in sarcoma in the United States: a focus on musculoskeletal lesions. Anticancer Res.

[3] Mordant P, Le Pimpec-Barthes F, Riquet M (2010). [Neurogenic tumors of the mediastinum in adults]. Rev Pneumol Clin.

[4] Takeda S, Miyoshi S, Minami M, Matsuda H (2004). Intrathoracic neurogenic tumors—50 years' experience in a Japanese institution. Eur J Cardiothorac Surg.

[5] Sukkarieh F, Van Meerhaeghe A, Delrée P, Brasseur P (2006). Tumeur maligne de la gaine des nerfs périphériques : une tumeur rare du médiastin postérieur. Rev Pneumol Clin.

[6] Wilson MP, Katlariwala P, Low G, Murad MH, McInnes MD, Jacques L, Jack AS (2021). Diagnostic accuracy of MRI for the detection of malignant peripheral nerve sheath tumors: a systematic review and meta-analysis. AJR Am J Roentgenol.

[7] Inci I, Soltermann A, Schneiter D, Weder W (2014). Pulmonary malignant peripheral nerve sheath tumour. Eur J Cardiothorac Surg.

[8] Ngabou UD, Mounguengui D, Owono Mbouengou JP (2014). [Intrathoracic giant peripheral nerve sheath tumor during Von Recklinghausen disease]. Rev Pneumol Clin.

[9] La Mantia E, Franco R, Cantile M, Rocco R, De Chiara A, Martucci N, Rocco G (2013). Primary intrapulmonary malignant peripheral nerve sheath tumor mimicking lung cancer. J Thorac Dis.

[10] Ferner RE, Gutmann DH (2002). International Consensus Statement on Malignant Peripheral Nerve Sheath Tumors in Neurofibromatosis. https://aacrjournals.org/cancerres/article/62/5/1573/509653/International-Consensus-Statement-on-Malignant.

[11] Sloan L, Terezakis SA, Blakeley JO, Slobogean B, Kleinberg LR (2018). Long-term outcomes of radiation therapy (RT) in the management of malignant peripheral nerve sheath tumors (MPNST) in patients with neurofibromatosis type 1 (NF1). Int J Radiat Oncol Biol Phys.

[12] Kahn J, Gillespie A, Tsokos M (2014). Radiation therapy in management of sporadic and neurofibromatosis type 1-associated malignant peripheral nerve sheath tumors. Front Oncol.

[13] Wang D, Zhang Q, Eisenberg BL (2015). Significant reduction of late toxicities in patients with extremity sarcoma treated with image-guided radiation therapy to a reduced target volume: results of Radiation Therapy Oncology Group RTOG-0630 trial. J Clin Oncol.

[14] Kroep JR, Ouali M, Gelderblom H (2011). First-line chemotherapy for malignant peripheral nerve sheath tumor (MPNST) versus other histological soft tissue sarcoma subtypes and as a prognostic factor for MPNST: an EORTC soft tissue and bone sarcoma group study. Ann Oncol.

